# Extraction and Purification of a Melanin from *Aureobasidium melanogenum* with Evaluation of Its Stability and In Vitro Antitumor Activity

**DOI:** 10.3390/microorganisms14081859

**Published:** 2026-08-20

**Authors:** Yu Lin, Wei Liang, Bo Yu, Lan Ma, Lin Shu, Zhiqun Liang, Wei Zeng

**Affiliations:** 1 Key Laboratory of Cell and Gene Therapy for Regional High-Incidence Diseases, Education Department of Guangxi Zhuang Autonomous Region, College of Basic Medical Sciences, Guilin Medical University, 1 Zhiyuan Road, Guilin 541199, China; 2 Guangxi Key Laboratory of Multimodal Biomarkers and Precision Diagnosis, Key Laboratory of Medical Biotechnology and Translational Medicine, Education Department of Guangxi Zhuang Autonomous Region, Guilin Medical University, 1 Zhiyuan Road, Guilin 541199, China; 3 College of Life Science and Technology, Guangxi University, 100 Daxue Road, Nanning 530004, China; 4 College of Artificial Intelligent Medicine, Guilin Medical University, 1 Zhiyuan Road, Guilin 541199, China

**Keywords:** melanin, *Aureobasidium melanogenum*, natural pigment, extraction, stability, antitumor activity

## Abstract

Microbial melanins are natural pigments with broad application potential, and *Aureobasidium melanogenum* is a promising strain for industrial production of melanin. However, the separation, purification, and biological activities of melanin derived from this strain remain largely unexplored. In this study, an efficient extraction and purification process for melanin from the fermentation broth of *A. melanogenum* GXZ-6 was established through single-factor optimization, and the final purity of the melanin reached 94.4%. Spectroscopic stability tests showed that the melanin was highly stable under dark, natural light, a wide pH range (2–12), and temperatures ≤ 37 °C. However, UV light, high temperature (>50 °C), ultrasonication, and certain metal ions (especially Cr^3+^) markedly altered its absorbance at 225 nm. More importantly, the purified melanin exhibited concentration-dependent antiproliferative activity against human cancer cell lines of A549, HCT116, MCF-7, and HepG2, with IC_50_ values ranging from 25.50 to 72.33 μg/mL. HepG2 cells were the most sensitive (IC_50_ = 25.50 μg/mL), and the melanin showed moderate selectivity toward normal hepatocytes (LO2). Mechanistic studies revealed that melanin effectively inhibited HepG2 cell migration, induced apoptosis, depolarized the mitochondrial membrane potential, and activated the caspase-3/PARP pathway. In summary, this study provides a simple, scalable, and environmentally friendly process for producing high-purity melanin with potential antitumor activity.

## 1. Introduction

Melanin is a class of natural pigments with complex polymeric structures derived from phenolic or indolic precursors. Based on the differences in their synthesis pathways and structures, there are several types such as eumelanin, pheomelanin, allomelanin, pyomelanin, and neuromelanin [1]. These melanins are widely distributed in microorganisms, plants, and animals, playing crucial roles in protection against UV radiation, oxidative stress, and heavy metal toxicity [2,3]. In recent decades, melanins have gained increasing attention for their diverse biological activities, including antioxidant, antitumor, immunomodulatory, radioprotective, and metal ion chelation. These unique physicochemical and biological properties make melanin a highly promising biomaterial for applications in pharmaceuticals, cosmetics, and environmental protection [4,5,6].

Compared with animal- or plant-derived melanin, microbial fermentation offers distinct advantages, including shorter production cycles, independence from seasonal and geographical constraints, and easier process control and scale-up [1,2,3]. At present, a variety of microorganisms have been reported to produce melanin, such as *Actinomycetes* [7], *Aspergillus* [8], *Armillaria* [9], *Aureobasidium* [10], *Auricularia* [11], *Bacillus* [12], *Brevundimonas* [13], *Pseudomonas* [14], *Streptomyces* [15]. Among these melanin producers, the yeast-like fungus *Aureobasidium melanogenum* is particularly attractive because of its robust growth on simple media, relatively high melanin titer, and simple fermentation process [16]. However, reports on the melanin production by *Aureobasidium* fermentation are still relatively limited.

We previously isolated *A. melanogenum* GXZ-6 from fresh plant samples; this strain produces a eumelanin containing an indole structure [16,17]. In addition, a fermentation process using a simplified medium (containing only glucose, MgSO_4_·7H_2_O, and KCl) without pH control for highly efficient production of melanin was also developed [16]. However, an efficient, scalable, and environmentally friendly downstream process for recovering melanin from the fermentation broth is still lacking. Existing methods often rely on repeated alkali extraction and acid precipitation, but the precise conditions such as NaOH concentration, extraction time, precipitation solvent, and number of cycles are rarely systematically optimized [18]. Moreover, the stability of microbial melanin under various environmental stresses, such as light, temperature, pH, ultrasonication, and metal ions, has not been comprehensively evaluated for *A. melanogenum*-derived melanin. The biological properties of melanin, particularly its antitumor activity, have been reported in several studies, mainly using melanin from *Lachnum* [19] or *Streptomyces* [20,21]. Nevertheless, systematic comparisons on the antiproliferative effect of melanin on various tumor cell lines and studies on the related anti-tumor mechanisms are rare.

Based on the progress in microbial melanin research, particularly from *A. melanogenum*, and building on our previous work, this study aimed to: (i) optimize a simple, cost-effective, and environmentally friendly extraction and purification process for melanin from the fermentation broth of *A. melanogenum* GXZ-6; (ii) comprehensively evaluate the stability of the purified melanin under various environmental factors; and (iii) investigate its in vitro antitumor activity against several human cancer cell lines, focusing on the most sensitive cell line (HepG2) to elucidate the underlying pro-apoptotic mechanism. The results provide a foundation for the future development of this natural pigment as a functional ingredient or therapeutic agent.

## 2. Materials and Methods

### 2.1. Microorganism and Medium

*Aureobasidium melanogenum* GXZ-6 was used as the melanin producer, which was deposited in the China Center for Type Culture Collection with accession number CCTCC M 2017517 [17]. The seed medium for cultivating *A. melanogenum* GXZ-6 consisted of glucose (20 g/L), peptone (20 g/L), and yeast extract (10 g/L). The fermentation medium for melanin production consisted of glucose (120 g/L), MgSO_4_·7H_2_O (0.1 g/L), and KCl (0.5 g/L) [16].

### 2.2. Fermentation Conditions for Melanin Production by A. melanogenum GXZ-6

Melanin production by *A. melanogenum* GXZ-6 was carried out in a fermenter under conditions described in our previous study [16]. First, the strain was inoculated into a 250 mL Erlenmeyer flask containing 50 mL of seed medium and cultivated with shaking at 30 °C and 180 rpm for 48 h to obtain the first-order seed culture. Then, 50 mL of the first-order seed culture was transferred into a 3 L Erlenmeyer flask containing 250 mL of seed medium and cultivated under the same conditions for 36 h to obtain the second-order seed culture. Finally, the second-order seed culture was inoculated at 8% (*v*/*v*) into a 5 L fermenter containing 3.75 L of fermentation medium. Fermentation was conducted at 30 °C for 10 days with aeration at 0.5 vvm and agitation at 250 rpm. The pH of the fermentation broth did not require control during the entire process.

### 2.3. Extraction and Purification of Melanin

The basic procedure for extracting and purifying melanin from the fermentation broth was as follows. First, a precipitate containing both cells and melanin was obtained by centrifugation of 30 mL of fermentation broth at 10,619× *g* for 8 min. The precipitate was resuspended in 10 mL of 1.0 M NaOH solution. The suspension was sonicated at 380 W for 5 min using 3 s active pulses followed by 4 s cooling intervals, and then heated in boiling water for 30 min. Cell debris was removed by another centrifugation step at 10,619× *g* for 8 min, and the supernatant was retained. Next, 0.5 volume of 6 M HCl was added to the supernatant under continuous stirring to precipitate melanin. The melanin-containing precipitate was collected by centrifugation at 10,619× *g* for 8 min, washed with water by three cycles of centrifugation and redispersion, and finally lyophilized to obtain purified melanin.

Single-factor experiments were performed to optimize key parameters in the extraction and purification process, including the concentration (0.1–5.0 M) and volume (10–30 mL) of NaOH, ultrasonication duration (0–20 min), boiling water bath extraction time (0–40 min), the type and volume of the precipitation solvent (HCl, ethanol, methanol), and the number of acid precipitation cycles (1–3 times). The optimal conditions were determined based on the yield and purity of the final melanin product.

The purity of the purified melanin was determined using a standard curve-based UV-Vis spectrophotometric method. Briefly, 1 mg of the purified melanin sample was dissolved in 1 M NaOH to prepare a 1000 mg/L stock solution. The solution was then diluted n-fold, and the absorbance at 225 nm (the characteristic absorption maximum of eumelanin) was measured. The melanin concentration (C_x_, mg/L) was calculated by interpolating the absorbance value on a standard curve prepared with commercial melanin (M8631, Sigma-Aldrich, St. Louis, MO, USA) as the reference standard. The purity was then calculated as:

$$Purity(\%)=\frac{n\times C_{x}}{1000}\times 100\%$$

Similarly, the recovery rate of the purification process was determined by comparing the melanin concentration before and after precipitation. The crude extract was diluted n-fold and its absorbance at 225 nm was measured to obtain C_1_ (mg/L). After precipitation with precipitant, lyophilization, and re-dissolution in an equal volume of 1 M NaOH, the purified melanin solution was diluted m-fold to obtain C_2_ (mg/L). The recovery was calculated as:

$$Recovery(\%)=\frac{m\times C_{2}}{n\times C_{1}}\times 100\%$$

### 2.4. Stability Test of Melanin

The stability of the purified melanin was evaluated by measuring changes in its absorbance at 225 nm under various conditions. All tests were performed using a 0.5 g/L melanin solution unless otherwise specified. For light stability, the melanin solution was exposed to natural light, ultraviolet light (254 nm, 15 W, distance 30 cm), or kept in the dark (covered with aluminum foil) at room temperature. Samples were taken at 0, 1, 2, 3, 6, 9, and 12 h for absorbance measurement. For temperature stability, the melanin solution was incubated at 4, 25, 37, 50, and 80 °C. Samples were taken at 0, 30, 60, 90, 120, 150, and 180 min, and then cooled to room temperature for absorbance measurement. For pH stability, the melanin solution was adjusted to different pH values (2, 4, 6, 8, 10, and 12) using 1 M HCl or 1 M NaOH, and then incubated at room temperature. Samples were taken at 0, 30, 60, 90, 120, 150, and 180 min for absorbance measurement. The absorbance was measured directly at the respective pH without readjustment. For ultrasonic stability, the melanin solution was subjected to ultrasonication (probe-type, 6 mm) at different power outputs (100, 150, 200, and 250 W) for 180 min, with 5 s on/5 s off pulses. Samples were taken at 0, 30, 60, 90, 120, 150, and 180 min for absorbance measurement. For metal ion stability, various metal chloride salts (Na^+^, K^+^, Ca^2+^, Zn^2+^, Fe^3+^, Cu^2+^, Mn^2+^, Mg^2+^, and Cr^3+^) were added to the melanin solution to a final concentration of 1 mM. A control sample without any metal ions was processed in parallel. After incubation at room temperature for 3 h, the solution was centrifuged at 10,619× *g* for 5 min, and the supernatant was used for absorbance measurement.

### 2.5. Evaluation of the In Vitro Antitumor Activity of Melanin

#### 2.5.1. Cell Lines and Culture Methods

Human lung cancer cells (A549), human cervical cancer cells (HeLa), human hepatocellular carcinoma cells (HepG2), and human normal hepatocytes (LO2) were kindly provided by Guangxi Medical University. Human breast cancer cells (MCF-7) and human colorectal cancer cells (HCT116) were purchased from the Cell Resource Center of Shanghai Institutes for Biological Sciences, Chinese Academy of Sciences.

A549, HepG2, HeLa, HCT116, and LO2 cells were cultured in RPMI 1640 medium (BC-M-017, Biochannel, Nanjing, China) supplemented with 10% fetal bovine serum (04-001-1ACS, BI, Haifa, Israel) and 1% penicillin-streptomycin solution (P1400, Solarbio, Beijing, China). MCF-7 cells were cultured in high-glucose DMEM medium (BC-M-005, Biochannel, China) supplemented with 10 μg/mL recombinant human insulin (P3378, Beyotime, Shanghai, China), 10% fetal bovine serum, and 1% penicillin-streptomycin solution. All cells were cultured in an incubator at 37 °C with 5% CO_2_.

#### 2.5.2. MTT Assay

The inhibitory effect of melanin on tumor cell proliferation was assessed using the MTT assay. Tumor cells (A549, HeLa, HepG2, MCF-7, and HCT116) and normal hepatocytes (LO2) in the logarithmic growth phase were harvested. After digestion with 0.25% trypsin (BC-CE-003, Biochannel, China), the cells were seeded into 96-well plates at a density of 5 × 10^3^ cells per well in 100 μL of medium. The plates were incubated for 24 h at 37 °C in a 5% CO_2_ incubator. The old medium was then discarded, and 100 μL of fresh medium containing varying concentrations (10, 20, 30, 40, 50, 60, 70, and 80 μg/mL) of melanin or the positive control 5-fluorouracil (5-FU, F476739, Aladdin, Shanghai, China) was added to each well. 5-FU, a first-line chemotherapeutic agent for hepatocellular carcinoma, was selected as the positive control due to its well-established clinical application, clearly defined mechanism of action (thymidylate synthase inhibition), and widespread use as a reference compound in in vitro antitumor studies of microbial natural products, enabling direct comparison with published data on other melanins. After 24 h of incubation, 20 μL of MTT solution (5 mg/mL, T6126, Macklin, Shanghai, China) was added to each well, followed by further incubation for 4 h. The supernatant was carefully aspirated, and 150 μL of DMSO was added to each well. The plates were then placed on a shaker and agitated at a low speed for 15 min to completely dissolve the formazan crystals. The absorbance of each well was measured at 570 nm using a microplate reader (Multiskan GO, Thermo Fisher, Waltham, MA, USA). Cell viability (%) was calculated using the following formula.

$$Cell\;viability\;(\%)=\frac{\left(A_{sample}-A_{background}\right)-A_{blank}}{A_{control}-A_{blank}}\times 100\%$$

where $A_{sample}$ is the absorbance of wells containing both cells and melanin, $A_{background}$ is the absorbance of wells containing the same concentration of melanin but without cells, $A_{blank}$ is the absorbance of wells containing neither cells nor melanin, and $A_{control}$ is the absorbance of wells containing cells but without melanin.

#### 2.5.3. Cell Scratch and Transwell Migration Assays

The effect of melanin on the migratory capacity of tumor cells was analyzed using cell scratch and Transwell migration assays.

Cell scratch assay: HepG2 cells in the logarithmic growth phase were harvested, digested with 0.25% trypsin, and seeded into 6-well plates at a density of 5 × 10^5^ cells per well in 2 mL of medium. The cells were cultured overnight at 37 °C in a 5% CO_2_ incubator until they reached 90–100% confluence. Prior to scratching, the medium was replaced with serum-free RPMI 1640 medium, and the cells were starved for 12 h. Two parallel scratches were made vertically across the bottom of each well using a sterile 10-μL pipette tip. The wells were then gently washed twice with PBS buffer to remove detached cells, and 2 mL of serum-free RPMI 1640 medium containing varying concentrations (0, 15, and 30 μg/mL) of melanin was added. The cells were observed and photographed under an inverted microscope (CKX41, Olympus, Tokyo, Japan) at 0, 24, and 48 h of incubation. The scratch area was measured using ImageJ (version 1.54r) software [22]. The wound healing rate (%) was calculated as (scratch area at 0 h − scratch area at indicated time)/scratch area at 0 h × 100%.

Transwell migration assay: HepG2 cells in the logarithmic growth phase were harvested, resuspended in serum-free RPMI 1640 medium, and adjusted to a density of 2 × 10^5^ cells/mL. A 200-μL of the cell suspension, supplemented with melanin at final concentrations of 0, 15, and 30 μg/mL, was added to the upper chamber of a Transwell insert (14341, Labselect, Beijing, China). The lower chamber received 600 μL of RPMI 1640 medium containing 10% FBS. The cells were incubated for 48 h at 37 °C in a 5% CO_2_ incubator. Subsequently, the inserts were removed, gently washed once with PBS buffer, and fixed with 4% paraformaldehyde at room temperature for 20 min. After discarding the fixative, the inserts were washed twice with PBS buffer and stained with 0.1% crystal violet solution at room temperature for 15 min in the dark. After two additional washes with PBS buffer, non-migrated cells remaining in the upper chamber were gently removed using a wet cotton swab. Cells were observed and photographed under an inverted microscope (CKX41, Olympus, Japan). For each chamber, five random fields were selected to count the total number of migrated cells on the lower surface, and the average was calculated.

#### 2.5.4. Hoechst 33342/PI Staining Assay

Hoechst 33342/PI dual staining was employed to assess the effects of melanin on tumor cell apoptosis. HepG2 cells in the logarithmic growth phase were harvested, digested with 0.25% trypsin, and seeded into 24-well plates at a density of 3 × 10^4^ cells per well in 1 mL of medium. The plates were incubated for 24 h at 37 °C in a 5% CO_2_ incubator. The old medium was discarded, and fresh medium containing varying concentrations (0, 15, and 30 μg/mL) of melanin was added to each well (final volume of 1 mL). After incubation for an additional 48 h, the medium was aspirated and the cells were gently washed twice with pre-cooled PBS buffer (pH 7.4). Next, 1 mL of PBS buffer containing Hoechst 33342 (5 μg/mL) and PI (5 μg/mL) was added to each well. After gently mixing and incubating at room temperature in the dark for 20 min, the staining solution was aspirated and the cells were washed twice with PBS buffer. Observation and image acquisition were performed using a fluorescence microscope (Imager Z1, Carl Zeiss, Oberkochen, Germany).

#### 2.5.5. Measurement of Mitochondrial Membrane Potential Using JC-1 Probe

The effect of melanin on the mitochondrial membrane potential of HepG2 cells was assessed using the JC-1 fluorescent probe. HepG2 cells in the logarithmic growth phase were seeded into 24-well plates at a density of 3 × 10^4^ cells per well in 1 mL of medium. After 24 h of culture at 37 °C in a 5% CO_2_ incubator, the medium was discarded, and 1 mL of fresh medium containing varying concentrations (0, 15, and 30 μg/mL) of melanin was added. After 48 h of incubation, the supernatant was aspirated, and the cells were gently washed once with pre-cooled PBS buffer. JC-1 staining working solution (BL711A, Biosharp, Beijing, China) was prepared according to the manufacturer’s instructions, and 1 mL was added to each well. After gentle mixing, the cells were incubated for 20 min at 37 °C in the dark. The staining solution was then aspirated, and each well was gently washed twice with 1 mL of pre-cooled JC-1 staining buffer. Finally, 1 mL of PBS buffer was added to each well, and the cells were immediately observed under a fluorescence microscope (Imager Z1, Carl Zeiss, Germany) and analyzed using a flow cytometer (Attune NxT, Invitrogen, Singapore).

#### 2.5.6. Western Blot Analysis of Apoptosis-Related Proteins

The effects of melanin on the expression of apoptosis-related proteins in HepG2 cells were investigated by Western blot. HepG2 cells were seeded into 24-well plates at a density of 3 × 10^4^ cells per well. After 24 h of incubation, the cells were treated for 48 h with medium containing varying concentrations (0, 15, and 30 μg/mL) of melanin. The cells were then harvested and washed twice with pre-chilled PBS buffer. RIPA lysis buffer (P0013B, Beyotime, China) supplemented with 1 mM PMSF was added, and the cells were lysed on ice for 30 min with vortexing every 10 min. The lysates were centrifuged at 12,000× *g* for 15 min at 4 °C, and the supernatants were collected. Protein concentrations were determined using a BCA Protein Assay Kit (P0010S, Beyotime, China).

The proteins were mixed with 5× SDS loading buffer at a 4:1 ratio and denatured in a boiling water bath for 10 min. The proteins were separated by 12% SDS-PAGE. Subsequently, the proteins were transferred to a methanol-activated PVDF membrane (0.45 μm, Millipore, Burlington, MA, USA) using the wet transfer system (70 V, 90 min) in ice-cold transfer buffer. The membrane was blocked with 5% non-fat dry milk in TBST at room temperature for 1 h. Primary antibodies, including rabbit anti-human caspase-3 (AB32351, 1:5000, Abcam, Cambridge, UK), rabbit anti-human cleaved PARP (AB32064, 1:1000, Abcam, UK), and mouse anti-human β-actin (AP0060, 1:5000, Bioworld, Visalia, CA, USA), were diluted in 5% BSA/TBST and incubated with the membrane overnight at 4 °C. After three washes with TBST, the membrane was incubated with HRP-conjugated secondary antibodies, including goat anti-rabbit IgG (AB6721, 1:5000, Abcam, UK) and goat anti-mouse IgG (AB97023, 1:5000, Abcam, UK), diluted in 5% non-fat dry milk/TBST, for 1 h at room temperature. Following three washes with TBST, the membrane was treated with ECL chemiluminescence substrate (P0018S, Beyotime, China) and visualized using a chemiluminescence imaging system (Tanon 5200, Tanon, Shanghai, China). Band intensities were analyzed using ImageJ (version 1.54r) software. The relative expression level of each protein was expressed as the ratio of the target protein band intensity to that of β-actin, and the control group (0 μg/mL melanin) was normalized to 1.

### 2.6. Statistical Analyses

All experiments were independently repeated at least three times, and each concentration was tested in triplicate. Data are presented as mean ± standard deviation (SD). Statistical comparisons among multiple groups were performed using one-way analysis of variance (ANOVA) followed by Waller-Duncan multiple range test using the SPSSAU Data Science Analysis Platform (https://spssau.com). A value of *p* < 0.05 was considered statistically significant. Graphs were generated using Origin 2021 (OriginLab Corporation, Northampton, MA, USA).

## 3. Results and Discussion

### 3.1. Optimization of Melanin Extraction from Fermentation Broth of A. melanogenum

Our previous studies have shown that *A. melanogenum* GXZ-6 can produce a eumelanin containing an indole structure [16]. To further investigate the biological activities of this melanin and lay a foundation for its future development and application, we first optimized the process for extracting melanin from the fermentation broth. Based on the alkali-solubility and acid-insolubility of melanin, we established an alkali extraction process to isolate crude melanin from the fermentation broth. Single-factor experiments were performed to optimize key extraction parameters using the yield of crude melanin (mg/mL fermentation broth) as the indicator.

**Figure 1:**
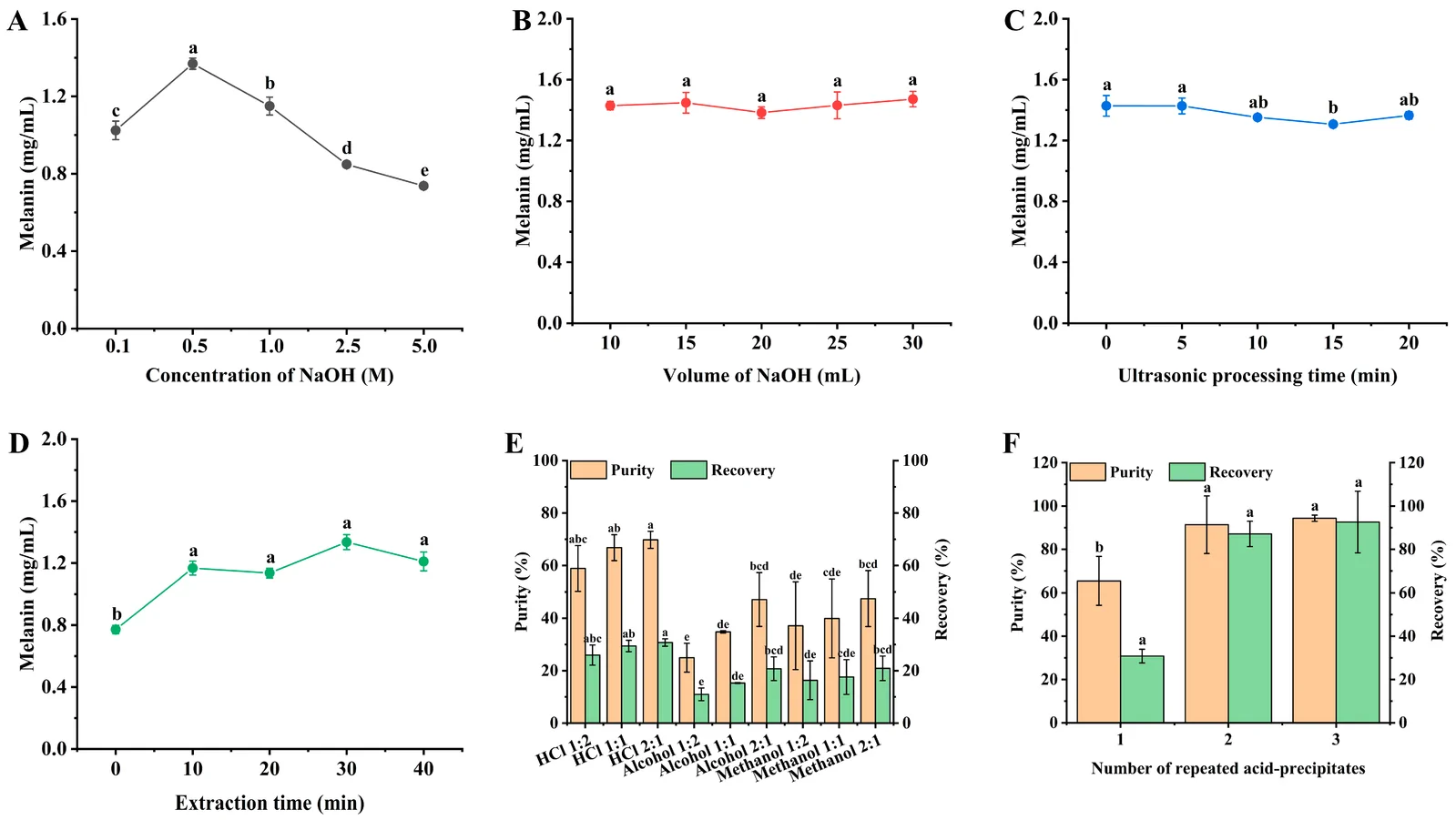
Optimization of melanin extraction and purification. (**A**) Effect of NaOH concentration on melanin yield (mg/mL broth). (**B**) Effect of NaOH volume per 10 mL broth. (**C**) Effect of ultrasonication duration. (**D**) Effect of boiling water bath time. (**E**) Comparison of precipitation solvents (6 M HCl, methanol, ethanol) at different volume ratios on the purity and recovery of melanin. (**F**) Effect of repeated HCl precipitation cycles on purity and recovery. Data represent mean ± SD (*n* = 3). Different letters indicate significant differences (*p* < 0.05).

As shown in Figure 1A, the optimal NaOH concentration for melanin extraction was 0.5 M. Lower (0.1 M) or higher (1.0–2.0 M) concentrations resulted in decreased yields. This decrease at higher alkali concentrations may be due to the co-extraction of more non-melanin impurities (e.g., polysaccharides and proteins) from the biomass, increasing the viscosity of the extract and interfering with the efficiency of centrifugation and subsequent acid precipitation [23]. Conversely, insufficient alkalinity may fail to fully solubilize the melanin from the fermentation broth. Therefore, 0.5 M NaOH was selected for subsequent experiments. Figure 1B demonstrates that varying the volume of 0.5 M NaOH from 10 to 30 mL per 10 mL of fermentation broth did not significantly affect the extraction yield. To minimize reagent consumption and downstream neutralization steps, a volume of 10 mL was chosen for the subsequent experiments.

Since melanin predominantly accumulates intracellularly (Figure S1), ultrasonication was tested to disrupt cells and facilitate melanin release. However, as presented in Figure 1C, ultrasonication for up to 20 min did not improve the extraction yield compared to no sonication (0 min). This suggests that the combination of alkali treatment and boiling is sufficient to extract melanin from *A. melanogenum*. Alkaline conditions can solubilize cell wall components, and the subsequent boiling further promotes cell lysis and melanin release. Therefore, ultrasonication was omitted from the procedure, simplifying the process and reducing energy consumption. Figure 1D shows that the extraction yield increased with prolonged heating in a boiling water bath, reaching a plateau after 10 min. No significant differences were observed among 10, 20, 30, and 40 min of heating (*p* > 0.05). The plateau may be attributed to a balance between continued melanin release and the onset of degradation or co-extraction of interfering impurities during extended boiling [23]. Therefore, 10 min of boiling was selected to save time and energy. The rapid release of melanin under alkaline boiling conditions is advantageous for large-scale production.

Based on these optimization results, the optimal extraction conditions were as follows: 10 mL of 0.5 M NaOH per 10 mL of fermentation broth, without ultrasonication, followed by boiling for 10 min. These conditions were used for all subsequent purification steps.

### 3.2. Purification of Melanin by Acid Precipitation

After alkali extraction, the crude melanin solution was subjected to precipitation to obtain a purer product. Because melanin is characteristically insoluble at low pH and in most organic solvents [24], we compared the precipitation efficiency of 6 M HCl, methanol, and ethanol at various volume ratios (precipitant:crude extract).

As shown in Figure 1E, HCl achieved higher purity and recovery than methanol or ethanol at all tested ratios. This may be because HCl not only lowers the pH to precipitate melanin but also helps remove non-melanin impurities that remain soluble under strongly acidic conditions. Among the tested ratios, a 2:1 (*v*/*v*) ratio of HCl to crude extract yielded the highest purity (69.8%) and recovery (30.7%). The relatively low recovery may be attributed to the partial solubility of melanin even under strongly acidic conditions, as its isoelectric point (pI) is typically in the range of 3.0–4.0 [25,26]. Consequently, some melanin may remain in the supernatant and be lost during the first precipitation. However, the high purity obtained with HCl justified its use for subsequent multi-cycle purification.

To further increase purity, the HCl precipitate was re-dissolved in 0.5 M NaOH and re-precipitated with the same volume of 6 M HCl. Figure 1F demonstrates that after the second precipitation, the purity increased markedly to 91.3%, and after the third precipitation, it reached 94.4%. The recovery rate remained above 85% for each precipitation step, indicating that the repeated dissolution-precipitation cycles are efficient and cause only modest product loss. This multi-cycle acid precipitation strategy is simple, scalable, and avoids the use of organic solvents, making it environmentally friendly. The final purity of 94.4% achieved with this simple three-cycle acid precipitation is at the lower end of the 95–98% range typical for commercial melanin standards. Thus, three cycles of HCl precipitation (2:1, *v*/*v*) were performed, and the final product was lyophilized to obtain purified melanin for subsequent experiments.

### 3.3. Stability of Melanin In Vitro

**Figure 2:**
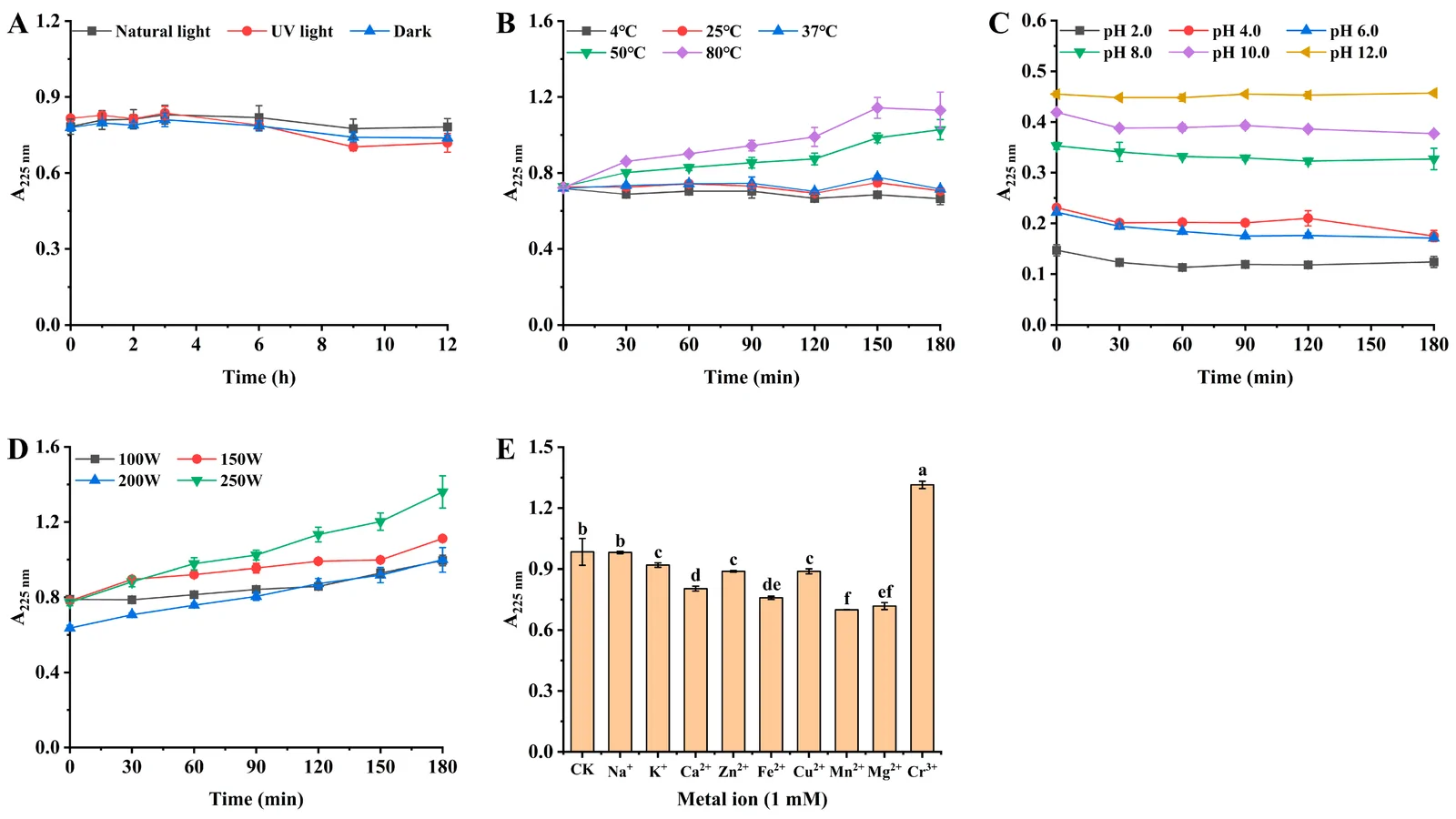
Stability of purified melanin under various conditions. (**A**) Light stability (natural light, UV light, dark). (**B**) Temperature stability (4–80 °C). (**C**) pH stability (pH 2–12). (**D**) Ultrasonication stability (100–250 W). (**E**) Metal ion stability (1 mM of indicated ions). Absorbance was measured at 225 nm. Data represent mean ± SD (*n* = 3). Different letters indicate significant differences (*p* < 0.05).

The purified melanin used in this study has been extensively characterized in our previous work, where it was identified as eumelanin containing an indole structure by SEM, UV-Vis, FTIR, ^1^H NMR, ^13^C NMR and XPS analyses [16]. To evaluate the stability of the purified melanin, we examined the effects of light, temperature, pH, ultrasonication, and metal ions on its absorbance at 225 nm. As shown in Figure 2A, the absorbance decreased only slightly and retained 94.7% of its initial value after 12 h under dark conditions, indicating good intrinsic stability. Natural light caused no significant change (*p* > 0.05). Under UV light, the absorbance initially increased slightly (0–3 h) and then decreased, retaining 88.1% after 12 h. The initial increase may be due to UV-induced aggregation or formation of new chromophores [27], while prolonged exposure leads to partial photodegradation [28]. Overall, the melanin exhibited excellent light resistance. As shown in Figure 2B, the absorbance remained stable over 180 min when the temperature was below 37 °C. However, the absorbance increased progressively at 50 °C and 80 °C. This is likely caused by disaggregation of melanin particles, increasing their solubility and apparent absorbance, or by thermally induced oxidation/polymerization generating additional chromophores [29,30]. Thus, the melanin is stable at routine temperatures but undergoes physicochemical changes at elevated temperatures.

As shown in Figure 2C, the initial absorbance increased with pH due to increased solubility of melanin in alkaline media. At each fixed pH, no significant change was observed over 180 min (*p* > 0.05), confirming that the melanin is stable across a wide pH range (2–12). As shown in Figure 2D, the absorbance gradually increased with time regardless of ultrasonication power (100–250 W). This suggests that ultrasonic cavitation disperses melanin aggregates into smaller particles or even molecularly dissolved species, enhancing light absorption [31]. Therefore, ultrasonication alters the physical state of melanin without causing degradation. As shown in Figure 2E, monovalent ions (Na^+^ and K^+^) had little effect on the absorbance. Divalent ions (Ca^2+^, Zn^2+^, Fe^2+^, Cu^2+^, Mn^2+^, and Mg^2+^) reduced absorbance, likely due to complexation of these metal ions with melanin’s catechol and carboxyl groups, leading to aggregation or precipitation [32]. Trivalent Cr^3+^ markedly increased absorbance, possibly forming a colored chromophore complex with strong absorption at 225 nm [33,34]. These findings highlight the potential of melanin for metal ion sensing or removal applications.

In summary, the melanin from *A. melanogenum* GXZ-6 is highly stable under most environmental conditions, with notable responses to UV light, high temperature, ultrasonication, and specific metal ions. These properties support its potential use in biotechnological and industrial applications.

### 3.4. Antitumor Activity of Melanin In Vitro

#### 3.4.1. Effect of Melanin on Tumor Cell Proliferation

**Figure 3:**
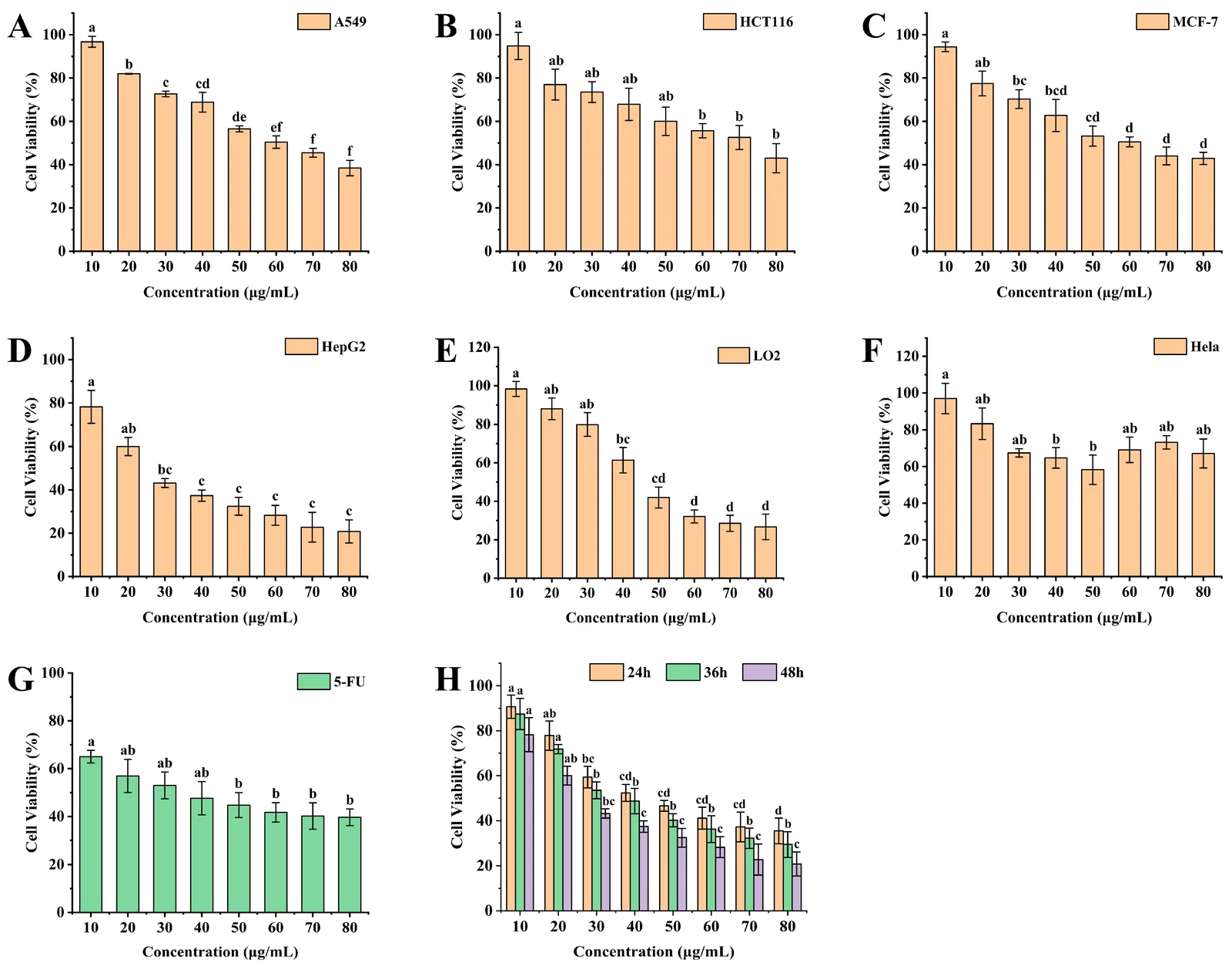
Antiproliferative activity of melanin against human cell lines (MTT assay). (**A**) A549, (**B**) HCT116, (**C**) MCF-7, (**D**) HepG2, (**E**) LO2, (**F**) HeLa, (**G**) 5-FU positive control on HepG2, (**H**) Concentration-dependent effect of melanin on HepG2 at 24, 36, and 48 h. Data represent mean ± SD (*n* = 3). Different letters indicate significant differences (*p* < 0.05).

The antiproliferative activity of purified melanin was evaluated by MTT assay. As shown in Figure 3A–E, melanin inhibited the proliferation of A549 (human lung cancer), HCT116 human colorectal cancer), MCF-7 (human breast cancer), HepG2 (human hepatocellular carcinoma), and LO2 (human normal hepatocytes) cells in a concentration-dependent manner. The calculated IC_50_ values were 61.68 ± 3.01 μg/mL (A549), 72.33 ± 6.04 μg/mL (HCT116), 60.85 ± 4.72 μg/mL (MCF-7), 45.61 ± 5.99 μg/mL (LO2), and 25.50 ± 3.43 μg/mL (HepG2). Among the cancer cell lines, HepG2 was the most sensitive to melanin. Interestingly, melanin exhibited an atypical concentration-response relationship for HeLa (human cervical cancer) cells (Figure 3F). The inhibition increased sharply at lower concentrations (10–30 μg/mL) but reached a plateau at concentrations above 40 μg/mL, where no further significant decrease in cell viability was observed. This cell line-specific plateau may be attributed to the unique biological characteristics of HeLa cells [35,36].

Notably, melanin suppressed only 18.15 ± 1.60% of normal hepatocytes (LO2) at the IC_50_ concentration for HepG2 (25.50 μg/mL), giving a selectivity index (SI = IC_50_ (LO2)/IC_50_ (HepG2)) of 1.79. The moderate selectivity indicates that melanin has a degree of preferential toxicity toward HepG2 cells over LO2, but further structural optimization or derivatization may be needed to improve its therapeutic window. Moreover, the positive control 5-fluorouracil (5-FU) showed an IC_50_ of 35.39 ± 4.47 μg/mL against HepG2 cells (Figure 3G), which is higher than that of melanin (25.50 μg/mL) under the same experimental conditions. However, the differences in drug uptake, metabolism, and mechanism of action may influence the direct comparison. Therefore, this comparison should be interpreted as a reference rather than a direct therapeutic equivalence. Furthermore, time-course experiments revealed that the inhibitory effect of melanin on HepG2 cells was time-dependent (Figure 3H). The IC_50_ values decreased from 43.81 ± 2.35 μg/mL at 24 h to 36.95 ± 4.62 μg/mL at 36 h and further to 25.50 ± 3.43 μg/mL at 48 h. In summary, melanin from *A. melanogenum* GXZ-6 demonstrated broad-spectrum antiproliferative activity against various human cancer cell lines, with the strongest effect on HepG2 cells. Its higher efficacy compared to 5-FU against HepG2, combined with lower toxicity toward normal hepatocytes, suggests that this melanin deserves further investigation as a potential antitumor agent, particularly for hepatocellular carcinoma.

#### 3.4.2. Effect of Melanin on HepG2 Cell Migration

**Figure 4:**
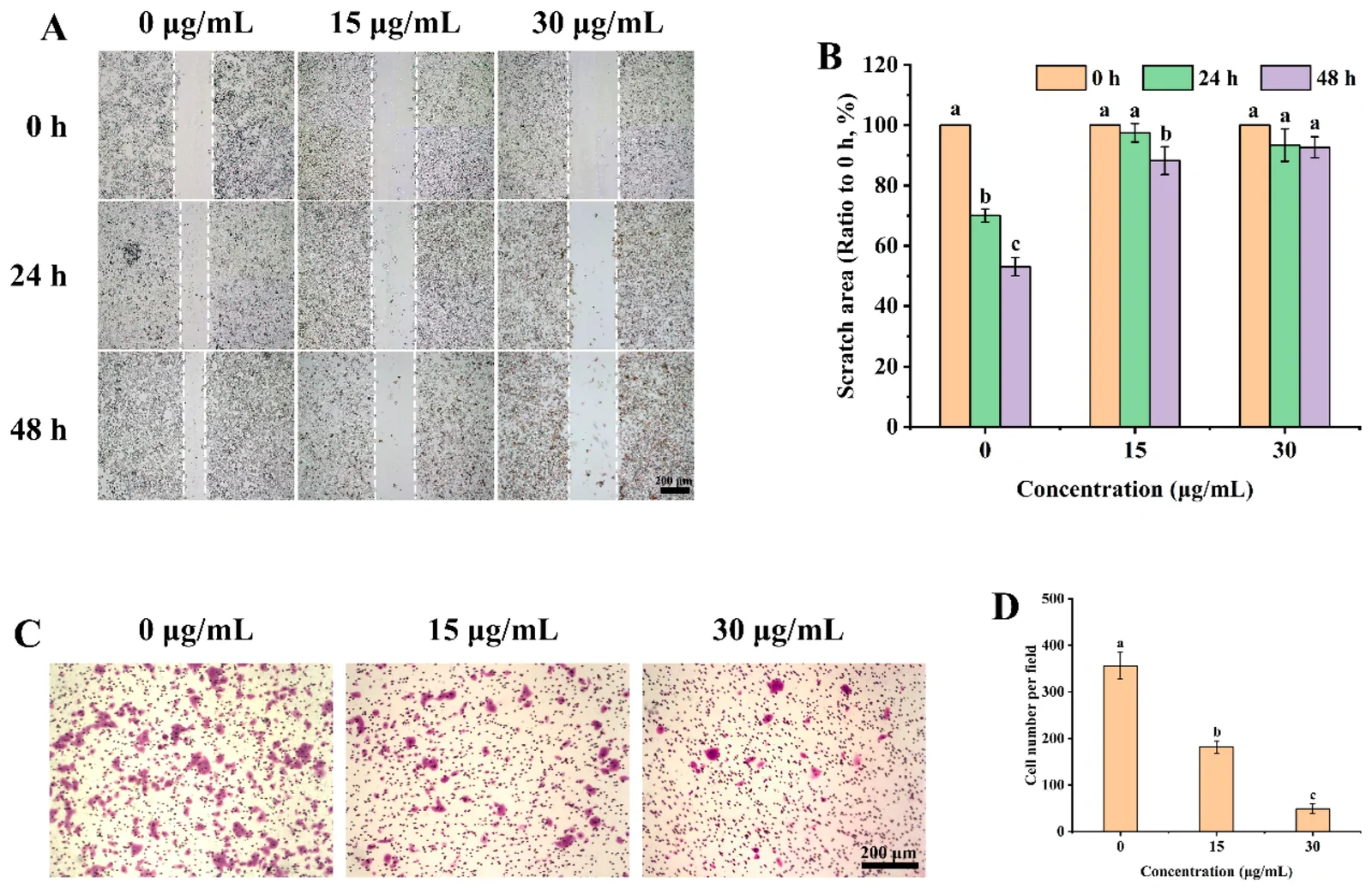
Effect of melanin on HepG2 cell migration. (**A**) Representative scratch wound images at 0, 24, and 48 h. (**B**) Quantification of remaining scratch area (normalized to 0 h). (**C**) Representative Transwell migration images. (**D**) Quantification of migrated cells per field. Data represent mean ± SD (*n* = 3). Different letters indicate significant differences (*p* < 0.05). Scale bar = 200 μm.

The effect of melanin on the migratory ability of HepG2 cells was evaluated using scratch wound healing and Transwell migration assays. As shown in Figure 4A,B, melanin inhibited the migration of HepG2 cells in a concentration-dependent manner. After 48 h of treatment, the remaining scratch area (normalized to 0 h) was 53.09 ± 3.05% in the control group (0 μg/mL), compared to 88.24 ± 4.61% and 92.68 ± 3.47% in the 15 and 30 μg/mL melanin groups, respectively. Furthermore, no significant difference was observed between 24 h and 48 h in the 30 μg/mL group, indicating that the maximal inhibitory effect on migration was already achieved within 24 h. In contrast, the control group showed a time-dependent decrease in scratch area. The Transwell assay (Figure 4C,D) further confirmed the anti-migratory effect of melanin. The number of migrated cells on the lower surface of the Transwell membrane decreased progressively with increasing melanin concentration. Quantitative analysis (Figure 4D) showed that the average number of migrated cells per field was 356.12 ± 28.93, 181.67 ± 13.01, and 49.25 ± 10.54 for 0, 15, and 30 μg/mL, respectively, with significant differences between groups. These results demonstrate that melanin significantly inhibits the migration of HepG2 cells. The anti-migratory effect was observed at concentrations (15–30 μg/mL) that are lower than the IC50 for proliferation inhibition (25.50 μg/mL at 48 h), suggesting a specific action on cell motility rather than a secondary effect of reduced viability.

#### 3.4.3. Effect of Melanin on HepG2 Cell Apoptosis

**Figure 5:**
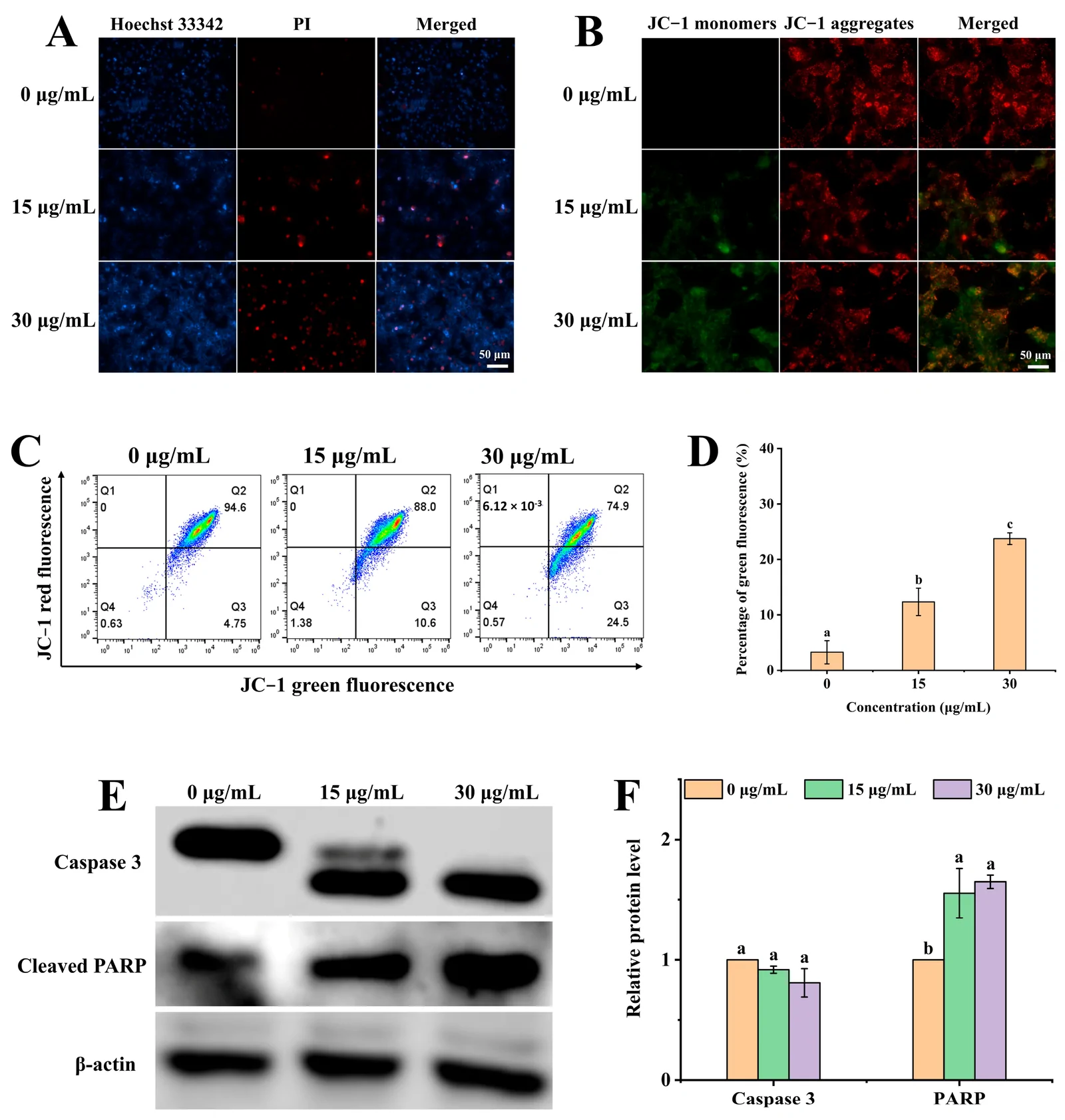
Effect of melanin on HepG2 cell apoptosis, mitochondrial membrane potential, and apoptosis-related protein expression. (**A**) Fluorescence microscopy images of Hoechst 33342/PI double staining for apoptosis detection. (**B**) Fluorescence microscopy images of JC-1 staining for mitochondrial membrane potential (ΔΨm). (**C**) Flow cytometry scatter plots of JC-1 staining for ΔΨm. (**D**) Quantification of cells with low ΔΨm. Data represent mean ± SD (*n* = 3). Different letters indicate significant differences (*p* < 0.05). (**E**) Western blot analysis of apoptosis related proteins. (**F**) Densitometric quantification normalized to β-actin. Data represent mean ± SD (*n* = 3). Different letters indicate significant differences (*p* < 0.05). Scale bar = 50 μm.

The apoptotic effect of melanin on HepG2 cells was evaluated using Hoechst 33342/PI dual staining. Hoechst 33342 is a cell-permeable DNA dye that stains all nuclei, with apoptotic cells displaying bright blue fluorescence due to chromatin condensation and fragmentation. Propidium iodide (PI) is impermeable to intact cell membranes and thus only enters cells with compromised membrane integrity (late apoptotic or necrotic cells). As shown in Figure 5A, control cells exhibited uniformly weak blue fluorescence with no red fluorescence, indicating normal nuclear morphology and intact cell membranes. After treatment with 15 μg/mL melanin, an increased number of cells showed bright blue fluorescence along with a few red-positive cells, suggesting the presence of apoptotic cells and some loss of membrane integrity. At 30 μg/mL melanin, more cells displayed bright blue and red fluorescence. These qualitative observations indicate that melanin induces apoptosis in HepG2 cells in a concentration-dependent manner. The induction of apoptosis by melanin aligns with the reduced cell viability observed in MTT assays. The appearance of PI-positive cells at higher concentrations suggests that prolonged or high-dose melanin treatment may lead to secondary necrosis or late apoptosis. Nevertheless, the present results provide direct microscopic evidence that melanin suppresses HepG2 cell growth at least partially through the induction of apoptosis.

#### 3.4.4. Effect of Melanin on Mitochondrial Membrane Potential of HepG2 Cell

The mitochondrial membrane potential (ΔΨm) of HepG2 cells was evaluated using the JC-1 fluorescent probe. JC-1 is a cationic dye that reversibly accumulates in mitochondria in a potential-dependent manner. At high ΔΨm, JC-1 forms red-fluorescent J-aggregates within the mitochondrial matrix; at low ΔΨm, it remains as a green-fluorescent monomer. Consequently, the red-to-green fluorescence ratio reflects the degree of mitochondrial depolarization [37]. HepG2 cells were treated with melanin (0, 15, and 30 μg/mL) and then stained with JC-1. As shown in fluorescence microscopy images (Figure 5B), control cells exhibited predominantly red fluorescence, with only minimal green signal. After melanin treatment, green fluorescence intensity increased, whereas red fluorescence decreased, indicating ΔΨm dissipation. This effect was concentration-dependent, with the most pronounced change observed at 30 μg/mL. Flow cytometry analysis (Figure 5C) validated these visual observations. The proportion of cells with low ΔΨm increased from 3.28 ± 2.03% in the control group to 12.35 ± 2.47% and 23.76 ± 1.05% in the 15 and 30 μg/mL melanin groups, respectively (Figure 5D). The loss of ΔΨm in HepG2 cells following melanin treatment is a hallmark of the intrinsic (mitochondrial) apoptotic pathway. These results are consistent with the morphological changes observed by Hoechst 33342/PI staining (chromatin condensation) and the concentration-dependent reduction in cell viability measured by the MTT assay. Collectively, our findings suggest that the antiproliferative effect of melanin on HepG2 cells is mediated, at least partially, through the induction of the mitochondrial apoptotic pathway.

#### 3.4.5. Effect of Melanin on Apoptosis-Related Protein Expression in HepG2 Cell

To further elucidate the molecular mechanism of melanin-induced apoptosis, the expression of pro-caspase-3 and cleaved PARP were examined by Western blot analysis. As shown in Figure 5E, treatment with melanin resulted in a concentration-dependent decrease in the level of caspase-3. Meanwhile, the level of cleaved PARP fragment increased progressively. Densitometric quantification (Figure 5F) confirmed these changes, with significant differences between control and melanin-treated groups.

PARP is a well-known substrate of caspase-3; its cleavage into two fragments of 89 kDa and 24 kDa is a hallmark of caspase-3-dependent apoptosis [38]. The observed decrease in caspase-3 together with increased cleaved PARP indicates that melanin activates the caspase-3-mediated apoptotic cascade. These results are consistent with the loss of mitochondrial membrane potential (Figure 5B) and morphological evidence of apoptosis (Figure 5A), supporting the conclusion that melanin induces apoptosis in HepG2 cells via the intrinsic mitochondrial pathway. Nevertheless, we acknowledge that the current evidence does not fully delineate the complete molecular cascade. Annexin V/PI flow cytometry and examination of Bcl-2 family proteins would provide more definitive evidence for apoptosis. These experiments, together with additional apoptosis-related proteins such as cleaved caspase-3, Bax, Bcl-2, cytochrome c, and caspase-9, will be addressed in our future mechanistic studies.

## 4. Conclusions

In this study, we developed a simple, cost-effective, and environmentally friendly process for the extraction and purification of melanin from *A. melanogenum* GXZ 6. The optimized method, which used 0.5 M NaOH extraction (10 mL per 10 mL broth), boiling for 10 min, and three cycles of HCl precipitation (2:1, *v*/*v*), yielded melanin with a purity of 94.4%. The purified melanin exhibited excellent stability under dark, natural light, a wide pH range (2–12), and temperatures ≤ 37 °C, while showing responsive changes at 225 nm to UV light, high temperature, ultrasonication, and trivalent chromium ions. In vitro antitumor assays revealed that the melanin selectively inhibited the proliferation of HepG2 hepatocellular carcinoma cells (IC_50_ = 25.50 μg/mL) with moderate selectivity over normal hepatocytes. Mechanistic studies demonstrated that melanin suppressed cell migration, induced apoptosis, depolarized the mitochondrial membrane potential, and activated the caspase-3/PARP pathway. Collectively, this work provides a robust downstream process for high-purity melanin production and comprehensively evaluates its stability profile and antitumor potential, laying the foundation for the subsequent development and application of this natural pigment. Future studies should focus on: (i) identifying the direct molecular target(s) of melanin through affinity chromatography or cellular thermal shift assay (CETSA); (ii) investigating the structure-activity relationship (SAR) through structural modifications; (iii) evaluating absorption, distribution, metabolism, and excretion (ADME) properties to assess drug-likeness; and (iv) validating the antitumor efficacy and safety in preclinical animal models. These investigations will be essential to advance this melanin toward clinical application.

## Data Availability

The original contributions presented in this study are included in the article/Supplementary Materials. Further inquiries can be directed to the corresponding author.

## Supplementary Materials

The following supporting information can be downloaded at: https://www.mdpi.com/article/10.3390/microorganisms14081859/s1, Figure S1: Time-dependent changes in the distribution of melanin produced by *Aureobasidium melanogenum* GXZ-6 between intracellular and extracellular fractions during fermentation.
