# Supplementary material for: Extraction and Purification of a Melanin from Aureobasidium melanogenum with Evaluation of Its Stability and In Vitro Antitumor Activity
Source: Microorganisms. 2026 Aug 20;14(8):1859. doi: 10.3390/microorganisms14081859 (PMC13515041; doi:10.3390/microorganisms14081859)
Supplement: Supplementary file 1 [file microorganisms-14-01859-s001.zip › microorganisms-4465172-supplementary.pdf]

Supplementary Data

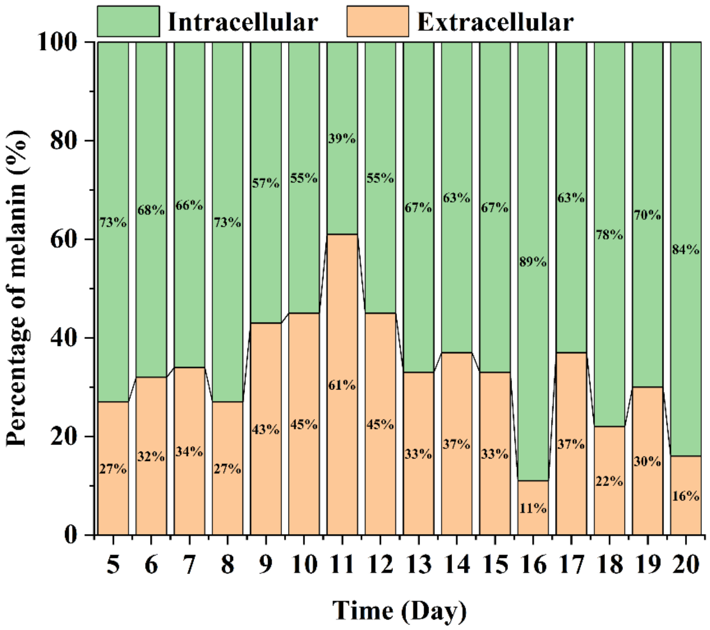

Figure S1. Time-dependent changes in the distribution of melanin produced by *Aureobasidium melanogenum* GXZ-6 between intracellular and extracellular fractions during fermentation.
